# Small Molecule-Assisted Exfoliation of Layered Zirconium Phosphate Nanoplatelets by Ionic Liquids

**DOI:** 10.1186/s11671-016-1559-6

**Published:** 2016-07-27

**Authors:** Fangqing Xia, Huaisong Yong, Xiao Han, Dazhi Sun

**Affiliations:** 1 Department of Materials Science and Engineering, South University of Science and Technology of China, Shenzhen, 518055 Guangdong Province China; 2 Shenzhen Key Laboratory of Nanoimprint Technology, South University of Science and Technology of China, Shenzhen, 518055 Guangdong Province China

**Keywords:** Nanoplatelets, Layered phosphates, Exfoliation, Intercalation, Ionic liquids

## Abstract

Exfoliation of layered inorganic nanomaterials into single-layered sheets has been widely interested in materials chemistry and composite fabrication. Here, we report the exfoliation of layered zirconium phosphate nanoplatelets by using small molecule intercalating agents in ionic liquids, which opens a new platform for fabricating single-layered inorganic materials from synthetic layered compounds.

## Background

How to obtain monolayer sheets from layered inorganic compounds has attracted dramatic attention in both scientific and technological communities [1–3]. Since the pioneering work on preparing single-layered graphene in 2004 [4], monolayer inorganic materials have many important emergency applications in various fields, such as lubricants [5–7], electronics [8, 9], sensors [10, 11], nanocomposites [12–16], and catalysis [17, 18]. Despite the direct growth of single-layered inorganic sheets [19, 20], exfoliation from layered crystalline inorganic compounds has been the main approach to produce these ultrathin, high-aspect-ratio, and 2D nanoscaled materials [1–3]. Many dry exfoliation methods have been developed and frequently used in literature, such as mechanical exfoliation [21], small-diameter low-boiling-point catalyst-assisted intercalated pyrolytic exfoliation route [22], indium-assisted vapor phase intercalated pyrolysis [23], and tin intercalant-assisted thermal cleavage [24]. On the other hand, exfoliation in liquid phase is a very promising and highly scalable approach for preparing high-quality 2D nanomaterials in mild conditions.

**Fig. 1:**
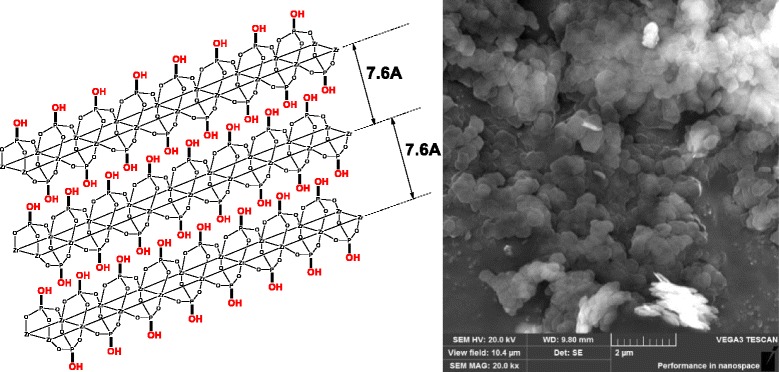
Chemical structure (*left*) and SEM image (*right*) of pristine layered ZrP nanoplatelets

Synthetic crystalline α-zirconium phosphate, Zr(HPO_4_)_2_·H_2_O (ZrP, structure is shown in Fig. 1), is often regarded as a model system for studying 2D layered compounds as it possesses well-designed structure, high purity, and controllable morphology [25]. Since the microstructure of ZrP has been fully investigated using high-resolution transmission electron microscopy [26] and atomic force microscopy [27], important crystal information for different kinds of ZrP can be obtained, e.g., thickness and growth directions within (001) planes (i.e., <110> and <100>). Although in the past intercalated ZrP materials have been widely prepared [28], methods that can exfoliate ZrP nanoplatelets into single-layered sheets are still very limited. Generally, to our best knowledge, two main approaches have been used to fully exfoliate ZrP nanoplatelets in liquids. One is to use strong organic bases (e.g., tetrabutyl ammonium hydroxide) in aqueous solutions to react with acidic ZrP platelets, and thus generate strong surface charges [15]. The other is the modification of ZrP nanoplatelets with polymeric surfactants (e.g., polyether amines) in solvents by a long-time ultra-sonicating treatment, which keeps the inorganic layers apart [29]. These wet exfoliation methods have been systematically reviewed by White and co-workers recently [30].

In this letter, we report a new method to exfoliate small molecule-intercalated ZrP nanoplatelets induced by ionic liquids. Although the addition of ionic liquids into ZrP nanoplatelets has been investigated [31, 32], the full exfoliation into single-layered sheets has not yet been studied in such system. This novel methodology opens a new route for achieving exfoliated morphology and may lead to new applications of layered phosphates and the related materials.

## Methods

### Materials

Zirconyl chloride (ZrOCl_2_·8H_2_O, 98 %, Aladdin reagents), phosphoric acid (85 %, CAS NO. is 13520-92-8, Aladdin reagents), 2-(2-aminoethoxy)ethanol (also called as diglycolamine, DGA, 98 %, CAS NO. is 929-06-6, Aladdin reagents), *n*-butylamine (NBA, 99.5 %, CAS NO. is 109-73-9, Aladdin reagents), *n*-hexylamine (HEA, 99 %, CAS NO. is 111-26-2, Aladdin reagents), 1-methyl-3-*n*-octylimidazolium bromide ([OMIm]Br, 98 %, CAS NO. is 61545-99-1, Aladdin reagents, it also could follow below procedure to synthesize it), and ethanol (99.5 %, CAS NO. is 64-17-5, Aladdin reagents) were used as received.

### Preparation of ZrP

A sample of 4.0 g ZrOCl_2_·8H_2_O was mixed with 40.0 ml 3.0 M H_3_PO_4_ and sealed into a Teflon-lined pressure vessel and heated at 200 °C for 24 h, respectively. The final products were identified as pristine ZrP. After the reaction, the products were washed by centrifugation for five times using deionized water. Then, the ZrP was dried at 80 °C for 24 h. The dried ZrP was ground with a mortar and pestle into fine powders.

### Preparation of the Used Ionic Liquid: 1-Methyl-3-*n*-octyl Imidazolium Bromide ([OMIm]Br)

74.574 g 1-methylimidazole (CAS No. is 616-47-7) and 100 ml anhydrous acetone were dissolved using a three-necked flask of 500 ml. The flask containing the 1-methylimidazole solution was placed into an oil bath at 85 °C with constant stirring under pure nitrogen condition. 192.969 g of *n*-octyl bromide (mole excess amount is about 10 %, CAS No. is 111-83-1) was added into the flask. The resulting solution was maintained at a constant temperature of 85 °C and constant stirring for 12~24 h. With reaction time proceeding, the reaction solution gradually became light-yellow. After reaction, the solvents in the solution were removed by vacuum rotary evaporation, and the viscous light-yellow liquid-like or slurry-like product was obtained. Then the viscous product was added into 1000 ml ethyl acetate; after intensive mixing, the nearly colorless-transparent viscous product was obtained, and the solvents in product were removed by vacuum rotary evaporation; this procedure was conducted repeatedly until the final viscous product becoming colorless-transparent, and the ultimate product was 1-methyl-3-*n*-octyl imidazolium bromide.

### Intercalation of ZrP Nanoplatelets

0.2 g of pristine ZrP with 25 g DGA/NBA/HEA/[OMIm]Br was mixed in a 50-ml glass bottle, respectively. The mixtures in the glass bottle were treated by ultra-sonication method (40 kHz) for 6 h at room temperature. After ultrasonic treatment, sample of mixtures were centrifugally washed by ethanol five times. Then, the washed product was dried at 60 °C and reduced pressure. The final product was identified as ZrP-DGA, ZrP-NBA, ZrP-HEA, and ZrP-[OMIm]Br, respectively.

### Small Molecule-Assisted Exfoliation of ZrP Nanoplatelets by [OMIm]Br

0.1 g ZrP-DGA/ZrP-NBA/ZrP-HEA with 25 g [OMIm]Br was mixed in a 50-ml glass bottle, respectively. The mixtures in the glass bottle were treated by ultrasound method (40 kHz) for 1 h at room temperature. After ultrasonic treatment, sample of mixtures were centrifuged at 14,000 rmp, and the final obtained gel-like precipitants were identified as ZrP-DGA-[OMIm]Br, ZrP-NBA-[OMIm]Br, and ZrP-HEA-[OMIm]Br.

### Characterization Methods

Scanning electron microscopy (SEM) studies were carried out using a TESCAN Electron Microscope (Vega3, The Czech Republic). Crystal structures of samples were analyzed by the X-ray diffraction (XRD) pattern obtained through a Rigaku X-ray diffractometer system (DMAX-2500, Japan). Transmission electron microscope (TEM) images were obtained with a Hitachi Transmission Electron Microscope (HT7700, Japan) operated at 80.0 kV. TEM samples were made by dispersing the powder (for exfoliation, gel-like precipitant was centrifugally washed by ethanol five times before dispersion) into ethanol. The alcohol suspensions were dropped onto copper grids and then allowed to dry in the air before the TEM imaging.

## Results and Discussion

**Fig. 2:**
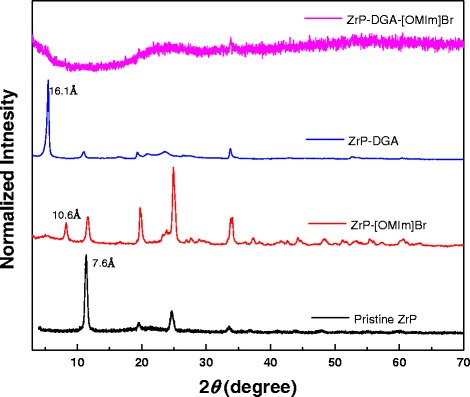
XRD patterns of ZrP nanoplatelets, ZrP-[OMIm]Br, ZrP-DGA, and ZrP-DGA-[OMIm]Br (from *bottom* to *top*)

ZrP nanoplatelets with an average diameter of ~400 nm were synthesized in water using a hydrothermal method as illustrated by Sun and co-workers [33] (also see the “Methods” section for details). The thickness of the synthetic ZrP is ~10.0 nm ± 3.0 nm based on Cheng and co-workers’ observation [26]. The analysis of the synthesized samples by scanning electron microscopy (SEM, Fig. 1) revealed a pseudo-hexagonal nanoplatelet structure. Crystalline ZrP nanoplatelets have an interlayer spacing of 7.6 Å, which is observed by the corresponding X-ray diffraction (XRD) pattern as illustrated in Fig. 2.

Pristine ZrP nanoplatelets were first treated in ionic liquid 1-methyl-3-*n*-octylimidazolium bromide ([OMIm]Br) by ultra-sonication. This sample is identified as ZrP-[OMIm]Br (see the “Methods” section for the detailed preparation). As observed in the corresponding XRD pattern in Fig. 2, the powder sample of ZrP[OMIm]Br shows additional diffraction peaks, indicating the mixture of pristine and intercalated ZrP nanoplatelets or partial intercalation of ZrP nanoplatelets with ionic liquids in the solid sample. The first peak shown in the corresponding XRD pattern indicates an interlayer spacing of 10.6 Å, about 3 Å larger than that of the pristine ZrP nanoplatelets. The increase of the interlayer spacing is the result of the intercalation of [OMIm]Br into ZrP layers. We further exposed the above sample to long-time sonication in additional [OMIm]Br; however, the XRD pattern remains unchanged, indicating the inefficiency of ionic liquids for intercalating ZrP nanoplatelets.

Pristine ZrP nanoplatelets were then treated with diglycolamine (DGA) by ultra-sonication. This sample is identified as ZrP-DGA (see the “Methods” section for the detailed preparation). The corresponding XRD pattern of the purified ZrP-DGA powder in Fig. 2 demonstrates the complete intercalation of the small amine molecules into ZrP layers. The sample was further exposed to long-time sonication in additional DGA; however, no exfoliation of ZrP nanoplatelets was found as expected. Linear long-chain polyether amines, e.g., commercial JEFFAMINE M-series, have been often used to intercalate ZrP nanoplatelets [14]. With long-time ultra-sonication and centrifugation, single-layered ZrP sheets can be separated from intercalated platelets; however, the yield of producing monolayer platelets is limited and the complete exfoliation cannot be realized.

**Fig. 3:**
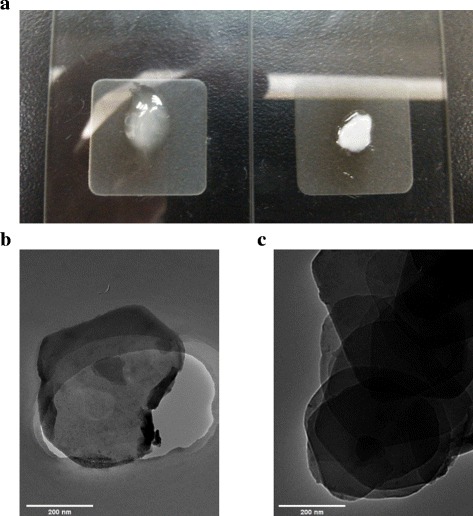
**a** Photo images of exfoliated ZrP-DGA-[OMIm]Br gel (*left*) and intercalated ZrP-DGA precipitate (*right*). **b** TEM image of exfoliated ZrP-DGA-[OMIm]Br. **c** TEM image of intercalated ZrP-DGA. *Scale bar* is 200 nm

DGA-intercalated ZrP nanoplatelets were next mixed with [OMIm]Br. After mild ultra-sonication for about 30–60 min, the sample turned transparent. Ultracentrifugation was then utilized to collect the products, and a clear yellowish gel-like sample was obtained (Fig. 3), which is designated as ZrP-DGA-[OMIm]Br. The corresponding XRD pattern in Fig. 2 shows no obvious diffraction peaks, indicating that a full exfoliation has been accomplished. The collected ZrP-DGA-[OMIm]Br gel can been easily redispersed in solvents, such as alcohol and acetone. The morphology of the exfoliated ZrP nanoplatelets was also studied by TEM (Fig. 3). The exfoliated thin nanosheets (Fig. 3b) appeared to be buckled slightly at the edge, possibly due to the sample shrinkage upon drying during the sample preparation. As for the intercalated sample (Fig. 3c), ZrP were obviously rigid and flat because of its large thickness.

The complete exfoliation of pristine ZrP nanoplatelets has been previously achieved by using tetrabutylammonium hydroxide (TBA-OH) in aqueous solution [12–16]. Firstly, the TBA-OH is a strong base, which can insert into the layers and reacts with the acidic ZrP nanoplatelets. The quaternary ammonium cations, TBA^+^, then absorb on the surface on ZrP nanoplatelets to general strong surface charges [12]. In aqueous solution, the electrostatic repulsion between TBA-absorbed ZrP nanoplatelets is strong enough to separate individual layers, thus achieving a full exfoliation. In this classic exfoliation case, both the choice of quaternary ammonium hydroxides and the type of solvents are important. Either larger quaternary ammonium hydroxides, e.g., tetrapentyl ammonium hydroxide, or smaller ones, such as tetrapropyl ammonium hydroxide are not able to exfoliate pristine ZrP nanoplatelets in water [12]. On the other hand, TBA-OH can only intercalate ZrP nanoplatelets in solvents, such as alcohol and acetone [14]. Therefore, TBA-OH is a very special agent for exfoliating ZrP nanoplatelets in water.

**Fig. 4:**
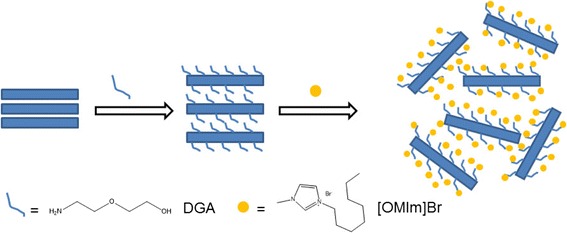
Exfoliation of DGA-intercalated ZrP nanoplatelets with ionic liquids (not drawn to scale)

In the current study, DGA, as a small molecule, is expected to only intercalate pristine ZrP nanoplatelets. With the aid of ionic liquid [OMIm]Br, the full exfoliation can be easily achieved. This could be due to the fact that ionic liquids are strong polar solvents, thus have a strong solvation effect on the DGA-modified ZrP nanoplatelets. The strong affinity between ionic liquid molecules and ZrP-DGA disturbs the layered structure and stabilizes the exfoliated single-layer sheets. This exfoliation process is illustrated in the cartoon shown in Fig. 4.

Ionic liquids have been used to exfoliate natural graphite into single-layered graphene with a high yield [2]. In this scenario, microwave irradiation was applied in the existence of certain oligomeric ionic liquid molecules even without using intercalating agents or surfactants. It was also found in the above work that HF-intercalation might help to achieve the microwave-induced exfoliation of graphite with some ionic liquids, which, otherwise, could not separate the layers alone in the same condition. Graphite layers are easy to slide apart due to the weak adhesive energy between the carbon sheets. Meanwhile, ionic liquids adhere to graphitic surface through a cation-π interaction. Therefore, with microwave irradiation or sonication, graphite layers can be exfoliated efficiently. However, in our work, ionic liquid molecules alone cannot exfoliate layered ZrP platelets as illustrated in Fig. 2 because ZrP nanoplatelets have a very strong interlayer adhesive energy owing to the strong H-bonding. The attraction of the ionic liquid cations to the hydroxyl groups on the ZrP nanoplatelet surface is unable to overcome the interlayer bonding energy, thus unable to even make an efficient intercalation. Similar attempts to directly intercalate bulky imidazolium-based ILs into ZrP in aqueous solution were also unsuccessful [34]. For *θ*-ZrP, due to the wider interlayer spacing (10.4 Å), ILs (BMIMCl) have intercalated into *θ*-ZrP layers successfully in Hu and co-workers’ work [35].

The introduction of DGA molecules into ZrP nanoplatelets is the key for achieving exfoliation in ionic liquids. The amine end of the DGA molecule is supposed to attach onto the ZrP nanoplatelets, similar to other amine molecules for the reaction and intercalation with ZrP platelets. The insertion of DGA into ZrP nanoplatelets breaks the H-bonding between pristine ZrP layers, expands the interlayer spacing, and weakens the interlayer adhesive energy. The rest of the DGA molecule is a small polar chain, which contains electron-rich ether and hydroxyl groups. Therefore, the ionic liquid cations are likely to go into the layers, interact with DGA through a cation-lone pair electron attraction, and then form highly charged surfaces, which leads to exfoliation of ZrP nanoplatelets and stabilization of the individual layers in ionic liquids.

**Fig. 5:**
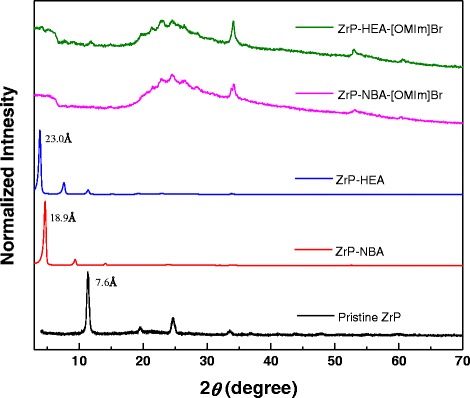
XRD patterns of ZrP nanoplatelets, ZrP-NBA, ZrP-HEA, ZrP-NBA-[OMIm]Br, and ZrP-HEA-[OMIm]Br (from *bottom* to *top*)

It is proposed that the polarity of the intercalating molecules affects the efficiency of the ZrP nanoplatelet exfoliation in ionic liquids. To test this hypothesis, *n*-butylamine (NBA) and *n*-hexylamine (HEA) were used to first intercalate ZrP nanoplatelets, which are designated as ZrP-NBA and ZrP-HEA, respectively. The intercalated samples were then mixed with [OMIm]Br under sonication. The experimental procedures were identical to the one used for DGA. The final products were identified as ZrP-NBA-[OMIm]Br and ZrP-HEA-[OMIm]Br, respectively (see the ESI† for the detailed experiments). As observed by XRD patterns shown in Fig. 5, powder samples of ZrP-NBA and ZrP-HEA have an interlayer spacing of 18.9 and 23.0 Å, respectively, indicating the intercalation of ZrP nanoplatelets with these two amines. The XRD patterns of both ZrP-NBA-[OMIm]Br and ZrP-HEA-[OMIm]Br show a small hump at the low diffraction angle region, indicating a not fully exfoliated, but disturbed layered structure. By comparing the XRD patterns of ZrP-NBA-[OMIm]Br (Fig. 5), ZrP-HEA-[OMIm]Br (Fig. 5), and ZrP-DGA-[OMIm]Br (Fig. 2), it is clear that DGA is more effective than both HEA and NBA on exfoliating ZrP nanoplatelets in ionic liquids. This phenomenon could be interpreted by the fact that both NBA and HEA have alkyl chains, which are less polar than DGA, thus have less affinity to the ionic liquid cations.

## Conclusions

In summary, exfoliation of layered ZrP nanoplatelets has been accomplished by using small molecule intercalating agents with high polarity in ionic liquids under mild sonication. Compared with other few existing exfoliation methodologies, our approach provides a new route for fabricating single-layered phosphate sheets in non-aqueous solutions with convenience and high efficiency. Future investigations will be focused on the use of such exfoliated ZrP nanoplatelets in composites, lubricants, and energy materials.
